# Mitochondrial Ultrastructure and Glucose Signaling Pathways Attributed to the Kv1.3 Ion Channel

**DOI:** 10.3389/fphys.2016.00178

**Published:** 2016-05-19

**Authors:** Christopher P. Kovach, Dolly Al Koborssy, Zhenbo Huang, Brandon M. Chelette, James M. Fadool, Debra A. Fadool

**Affiliations:** ^1^Program in Neuroscience, Florida State UniversityTallahassee, FL, USA; ^2^Department of Biological Science, Florida State UniversityTallahassee, FL, USA; ^3^Institute of Molecular Biophysics, Florida State UniversityTallahassee, FL, USA

**Keywords:** mitochondria, olfactory bulb, diet-induced obesity, glucose transporter, potassium channel

## Abstract

Gene-targeted deletion of the potassium channel Kv1.3 (Kv1.3^−∕−^) results in “Super-smeller” mice with a sensory phenotype that includes an increased olfactory ability linked to changes in olfactory circuitry, increased abundance of olfactory cilia, and increased expression of odorant receptors and the G-protein, G_olf_. Kv1.3^−∕−^ mice also have a metabolic phenotype including lower body weight and decreased adiposity, increased total energy expenditure (TEE), increased locomotor activity, and resistance to both diet- and genetic-induced obesity. We explored two cellular aspects to elucidate the mechanism by which loss of Kv1.3 channel in the olfactory bulb (OB) may enhance glucose utilization and metabolic rate. First, using *in situ* hybridization we find that Kv1.3 and the insulin-dependent glucose transporter type 4 (GLUT4) are co-localized to the mitral cell layer of the OB. Disruption of Kv1.3 conduction via construction of a pore mutation (W386F Kv1.3) was sufficient to independently translocate GLUT4 to the plasma membrane in HEK 293 cells. Because olfactory sensory perception and the maintenance of action potential (AP) firing frequency by mitral cells of the OB is highly energy demanding and Kv1.3 is also expressed in mitochondria, we next explored the structure of this organelle in mitral cells. We challenged wildtype (WT) and Kv1.3^−∕−^ male mice with a moderately high-fat diet (MHF, 31.8 % kcal fat) for 4 months and then examined OB ultrastructure using transmission electron microscopy. In WT mice, mitochondria were significantly enlarged following diet-induced obesity (DIO) and there were fewer mitochondria, likely due to mitophagy. Interestingly, mitochondria were significantly smaller in Kv1.3^−∕−^ mice compared with that of WT mice. Similar to their metabolic resistance to DIO, the Kv1.3^−∕−^ mice had unchanged mitochondria in terms of cross sectional area and abundance following a challenge with modified diet. We are very interested to understand how targeted disruption of the Kv1.3 channel in the OB can modify TEE. Our study demonstrates that Kv1.3 regulates mitochondrial structure and alters glucose utilization; two important metabolic changes that could drive whole system changes in metabolism initiated at the OB.

## Introduction

There are multifarious roles for potassium channels beyond being drivers of the resting potential (Kaczmarek, 2006), although classically potassium channels are dampeners of excitability through timing of the interspike interval and shaping of the action potential (AP; Jan and Jan, 1994; Yellen, 2002). Among the non-conductive functions of potassium channels lies their ability to detect, be regulated, or be modulated by energy substrates or metabolites, namely glucose, ATP, or NADPH (Pan et al., 2011; Tucker et al., 2013; Tinker et al., 2014). This original research article will present a review of metabolic sensing by a brain region outside the traditional endocrine axis controlling food intake and energy balance—the olfactory bulb (OB). We highlight the role of a particular potassium channel, Kv1.3, which is highly expressed in the OB and may serve as the molecular sensor of metabolism. We hypothesize that the olfactory system has a dual function, to transduce external chemical signals, or odorants, into an internal representation, and simultaneously to detect internal chemistry, namely metabolically important molecules, such as glucose and insulin (Fadool et al., 2000, 2011; Tucker et al., 2013). Thus, the OB is a detector of metabolic state; the biophysical properties of Kv1.3 are modulated by energy availability and the disruption of energy homeostasis can modify olfactory sensory coding (Palouzier-Paulignan et al., 2012). Such metabolic sensing could change food intake given the intimate relationship of olfactory perception to satiety and food choice (Aime et al., 2007; Tong et al., 2011; Badonnel et al., 2014; Soria-Gomez et al., 2014; Lacroix et al., 2015). We summarize current data leading to the glucose-sensing ability of Kv1.3-expressing neurons in the OB and present new data showing the expression of glucose transporter type 4 (GLUT4) in the OB. We report biochemical evidence that Kv1.3 activity can alter GLUT4 translocation, and demonstrate ultrastructural changes in the OB mitochondria of Kv1.3^−∕−^ mice. To place our new studies in context, we will (1) describe the phenotype of Kv1.3^−∕−^ mice and the role of this voltage-dependent channel in olfaction, (2) discuss the potential therapeutic benefits of targeting Kv1.3 channels centrally and peripherally for energy homeostasis, (3) present the known distribution and role of glucose transporters in the OB, (4) summarize the known expression of Kv1.3 in mitochondria, and (5) conclude the introduction by describing recent data that demonstrate the impact of metabolic imbalance on the structure/function of the OB.

The discovery of the many non-conductive roles for Kv1.3 was made through loss of function studies using a whole-animal, targeted deletion of the Kv1.3 gene (Koni et al., 2003). We and other laboratories identified the Kv1.3^−∕−^ mice as thinner than their wild-type (WT) counterparts without caloric self-restriction (Xu et al., 2003, 2004; Fadool et al., 2004). The mice had lower plasma fasting glucose levels, lower leptin levels, and had greater insulin sensitivity when challenged with an intraperitoneal glucose tolerance test (Xu et al., 2003; Tucker et al., 2008, 2010; Thiebaud et al., 2014). Using a custom-designed metabolic chamber to quantify systems physiology parameters and ingestive behaviors (Williams et al., 2003), we found that the Kv1.3^−∕−^ mice ate frequent small meals and drank large boluses of water while still maintaining identical total caloric and water intake as that of WT animals (Fadool et al., 2004). Kv1.3^−∕−^ mice showed slightly elevated metabolic activity (Xu et al., 2003; Fadool et al., 2004) and increased locomotor activity, particularly in the dark cycle (Fadool et al., 2004). Interestingly, Hennige et al. (2009) demonstrated that an i.c.v. injection of the Kv1.3 pore blocker, margatoxin, similarly elevated locomotor activity and increased cortical AP frequency. Recently when provided access to voluntary running wheels, the Kv1.3^−∕−^ mice had no marked irregularity in circadian rhythm. The mice ran the same daily distance as WT mice, but they ran at a lower velocity and in more frequent exercise bursts (Gonzalez et al., unpublished data). When examined for olfactory acuity, Kv1.3^−∕−^ mice showed a heightened sense of smell in terms of both odorant discrimination and odor threshold (Fadool et al., 2004). The Kv1.3 channel carries 60–80% of the outward current in mitral cells of the OB (Fadool and Levitan, 1998), which are the first order processing cells for olfactory information. Not unexpectedly, deletion of Kv1.3^−∕−^ in these neurons elicits an increase in the evoked firing frequency, attributed to more spikes/AP cluster, a shortened interburst interval, and also a depolarizing shift in the resting membrane potential (Fadool et al., 2004, 2011). The olfactory circuitry of Kv1.3^−∕−^ mice has a smaller number of axonal projections from the olfactory epithelium to identified synaptic targets, or glomeruli, and the projection is not mistargeted. The glomeruli, however, are smaller and supernumerary (Biju et al., 2008). Finally, the expression of G-protein coupled odor receptors (ORs) is enhanced in the Kv1.3^−∕−^ mice, with a concomitant increase in OR density in the ciliary processes that contain this odorant transduction machinery (Biju et al., 2008). This collective phenotype in the knockout mice reveals the broad span of non-conducting functions of Kv1.3 channels, from energy homeostasis, to ingestive behavior, to axonal targeting, and sensory perception or odor tuning.

Because Kv1.3^−∕−^ mice exhibited altered ingestive behaviors (Fadool et al., 2004), we and others challenged them with modified-fat diets and discovered a resistance to diet-induced obesity (DIO) (Xu et al., 2003; Tucker et al., 2012). Moreover, when Kv1.3^−∕−^ mice were bred to homozygosity with melanocortin-4 receptor-null mice (Tucker et al., 2008), which have a disruption of the hypothalamic, anorexogenic pathway and are a model for genetic obesity, the resultant progeny had reduced adiposity and body weight, attributed to increased locomotor activity and energy expenditure. Interestingly, while Kv1.3 has a selective distribution within the central nervous system (dentate gyrus, OB, and olfactory cortex; Kues and Wunder, 1992), its biophysical properties were first characterized in T lymphocytes (Cahalan et al., 1985). Others have explored the channel's capacity to regulate either body weight or insulin sensitivity (Xu et al., 2004; Upadhyay et al., 2013) by blocking channel activity in the periphery. Intraperitoneal injection of margatoxin, which blocks the vestibule of the Kv1.3 channel (Knaus et al., 1995), was found to increase insulin sensitivity in wildtype and genetic models of obesity, namely ob/ob and db/db mice (Xu et al., 2004). A similar effect was discovered as a result of a different channel blocker, ShK186, which was effective in increasing peripheral insulin sensitivity in mice that were fed an obesogenic diet of high fat and fructose (Upadhyay et al., 2013). Beyond enhanced insulin sensitivity in DIO mice, ShK-186 treatment also reduced weight gain, adiposity, and fatty liver and activated brown fat to augment oxygen consumption and energy expenditure. Although there have been conflicting reports on the ability of the channel to improve insulin sensitivity based upon the pharmacological inhibition of peripheral Kv1.3 across different skeletal muscle cell lines (Straub et al., 2011; Hamilton et al., 2014) and exploration of the selectivity of Kv1.3 blockers themselves (Bartok et al., 2014), peripheral targeting of the Kv1.3 channel remains a strong therapeutic target for regulating diabetes, inflammation, and other diseases (Choi and Hahn, 2010; Pérez-Verdaguer et al., 2015).

One manner by which Kv1.3 is thought to promote insulin sensitivity and glucose uptake is through the insulin-sensitive glucose transporter type 4 (GLUT4) (Xu et al., 2004). Inhibition or gene-targeted deletion of Kv1.3 stimulates glucose uptake and translocation of GLUT4 in adipose tissue and skeletal muscle. Because glucose (C_6_H_12_O_6_) is a polar molecule, its transport across the plasma membrane requires integral transport proteins. Carriers for glucose were first isolated from the membranes of HepG2, a hepatic cell line, as reviewed by Baly and Horuk (1988). Since then, different families and classes of glucose transporters have been identified in peripheral tissues as well as in the brain. Glucose is transported across biological membranes via glucose transporters (GLUTs) according to “an alternating conformer model.” According to this model, glucose can bind to one of two mutually-exclusive binding sites of the transporter, either on the extracellular or the intracellular site. The glucose transporter switches from one conformation to another to release its substrate (Carruthers, 1990). GLUTs comprise 14 family members named in the order they were cloned, and divided into three subfamilies: class I (GLUT1-4, and GLUT14), class II (GLUT5, GLUT7, GLUT9, and GLUT11), and class III (GLUT6, 8, 10, 12, and the myoinositol transporter HMIT1 or GLUT13) (see Table 1). GLUTs exhibit diverse substrate and tissue specificity, resulting in distinct functional characteristics. Their sequences, however, are similar in that (1) 12 helices span the plasma membrane with seven conserved glycine residues in each helix, (2) the intracellular surface has several basic and acidic residues, and (3) two tryptophan and two tyrosine residues are conserved (Joost and Thorens, 2001).

**Table 1:** **Distribution of glucose transporters and Kv1.3 channel**.

	**Transporter/Channel**	**Experimental Model**	**Technique**	**Localization**	**References**
CLASS I	GLUT1	Mouse	IHC (gold particles)	Asymmetric expression in cortex, hippocampus, cerebellum, and OB	Dobrogowska and Vorbrodt, 1999
	GLUT1 and GLUT2	Mouse	IHC and *in situ* hybridization	Tanycytes in hypothalamus	García et al., 2003
	GLUT1, GLUT3, and GLUT4	Bovine, Rat	Western blot, qPCR, and ICC	Monocytes, olfactory bulb	O'Boyle et al., 2012; Al Koborssy et al., 2014
CLASS II	GLUT 5	Rat	RNA blotting	Small intestine, kidney, and brain	Rand et al., 1993
	GLUT7	Human, Rat	Northern blot, IHC, and Western blot	Small intestine, colon, testes, and prostate in humans. Small intestine and low expression in colon of rat	Cheeseman, 2008
	GLUT9	Human	Northern blot	Highly expressed in kidney and liver. Low levels of expression in lung, leukocytes, & heart	Phay et al., 2000
	GLUT11	Human, *Xenopus* oocytes	Northern blot and qPCR	Three splice variants: GLUT11-A in heart, skeletal muscle and kidney. GLUT11-B in kidney, adipose, and placenta. GLUT11-C in adipose, heart, skeletal muscle, and pancreas.	Scheepers et al., 2005
CLASS III	GLUT10 and GLUT12	Human, Mouse	RT-PCR	Adipose tissue: mature adipocytes and stromal vascular cells	Wood et al., 2003
	GLUT8	Mouse, Rat, Human	Northern blot	Highly expressed in human testes and sperm cells. Low amounts detected in several brain areas, skeletal muscle, heart, and small intestine	Schmidt et al., 2009
	GLUT13 (HMIT1)	Mouse, *Xenopus* oocytes	Western blot, Northern blot, Immunofluorescence	Multiple brain areas; mostly in glial cells but also present in neurons	Cura and Carruthers, 2012
CHANNEL	Kv1.3	Human cell line	Patch-clamp electrophysiology	Voltage-gated potassium channel first characterized in T-lymphocytes	Cahalan et al., 1985
	Kv1.3	Rat	*In situ* hybridization	Dentate gyrus, OB, piriform cortex	Kues and Wunder, 1992
	Kv1.3	Human and mouse cell lines	Western blot	T-lymphocytes	Cai and Douglass, 1993
	Kv1.3	Rat cell culture, Mouse, Rat	ICC and Patch-clamp electrophysiology	Kv1.3 highly expressed in mitral cells of olfactory bulb	Fadool and Levitan, 1998; Fadool et al., 2000, 2004
	Kv1.3	Rat	IHC, RT-PCR, immunofluorescence, and immunoblotting	Kv1.3 expressed in both cortex and medulla of kidney	Carrisoza-Gaytán et al., 2010

IHC, immunohistochemistry; ICC, immunocytochemistry; OB, olfactory bulb; RT-PCR, reverse transcriptase PCR; qPCR, quantitative PCR.

It has been shown that loss of Kv1.3 translocates GLUT4, a member of the family of facilitative glucose transporters (GLUTs) or class I, in a calcium-dependent and insulin-independent manner (Xu et al., 2004; Desir, 2005). In adipocytes, the mechanism of translocation is thought to occur through a PI3K-independent pathway consisting of depolarizing the plasma membrane and increasing the intracellular Ca^2+^ concentration (Li et al., 2006). The co-localization of GLUT4 and Kv1.3 centrally has not been explored and the mechanism of glucose signaling in the OB has only recently been investigated (Tucker et al., 2013; Al Koborssy et al., 2014) for such an energy demanding process as odor processing (Carter and Bean, 2009; Lecoq et al., 2009, 2011). Kv1.3 currents can be modulated by metabolically active (d-glucose) rather than inactive (l-glucose) glucose in a dose-dependent manner that follows a bell-shaped curve (Tucker et al., 2013). Using a slice preparation of the OB and current-clamp configuration, we found that mitral cells of the OB could be either excited or inhibited by glucose (Tucker et al., 2013). The change in mitral cell excitability was linked to a changed latency to first spike and alternation of the AP cluster length. Kv1.3^−∕−^ mice were insensitive to glucose and unlike modulation by phosphorylation, glucose-induced change in mitral cell activity was rapid and reversible within the time course of a patch recording. We now report the presence of GLUT4 and Kv1.3, centrally, co-localized in the mitral cell layer of the OB. Moreover, using a heterologous expression system (HEK 293 cells), we are able to increase the translocation of GLUT4 when the pore of Kv1.3 (Delaney et al., 2014) is mutated to yield a non-conducting channel. This suggests that a number of modulators of Kv1.3 that can inhibit channel conductance (src, EGF, insulin, BDNF, glp-1, Nedd4-2) (Holmes et al., 1996; Bowlby et al., 1997; Fadool et al., 1997, 2000; Fadool and Levitan, 1998; Cook and Fadool, 2002; Colley et al., 2009; Thiebaud et al., 2016; Velez et al., 2016), could be poised to increase GLUT4 translocation and the utilization of glucose by mitral cells of the OB.

While Kv1.3 channels do not exist in isolation in the plasma membrane—they are part of a well-characterized scaffold of proteins and adaptor proteins that act to interface signaling cascades with the channel (Cook and Fadool, 2002; Marks and Fadool, 2007; Colley et al., 2009)—the channels are not solely restricted to this organelle. The channel is additionally highly expressed in the inner mitochondrial membrane, or IMM, of several cell types including lymphocytes (Szabo et al., 2005), cancer cells (Leanza et al., 2012), and hippocampal neurons (Bednarczyk et al., 2010). It is for this reason, and given the metabolic and olfactory phenotypes of Kv1.3^−∕−^ mice, that we undertook an ultrastructural analysis of mitochondria in the OB. The IMM is home to many types of ion channels and exchangers for a variety of cations and anions (Bernardi, 1999), which maintain a negative membrane potential of the organelle. The tightly controlled IMM permeability is critical for efficient ATP production and a dysregulation of IMM permeability often leads to cell death (Kim et al., 2003). It has been proposed that potassium ion channels play an important role in the control of the integrity of the IMM (Szewczyk et al., 2009) whereby substantial evidence has shown cardio- and neuro-protective effects of targeting mitochondrial potassium channels (Szewczyk and Marban, 1999; Busija et al., 2004; O'Rourke, 2004). The mechanism of these effects has been linked to the mitochondrial production of reactive oxygen species or ROS (Malinska et al., 2010). Interestingly, it has been shown that oxygen/glucose deprivation of microglia results in suppression of Kv1.3 currents in a tyrosine phosphorylation signaling cascade, which can be significantly attenuated by ROS scavengers (Cayabyab et al., 2000). It is possible that mitochondrial Kv1.3 functions could go beyond cytoprotection and involve ROS-dependent energy metabolism. ROS signaling is usually considered to be detrimental and damage-promoting, however, ROS can act as signaling molecules regulating organismal homeostasis, stress responsiveness, health, and longevity (Shadel and Horvath, 2015). ROS production is linked to the central control of whole body metabolism (Shadel and Horvath, 2015). In fact, there is considerable evidence showing that ROS can enhance insulin sensitivity by oxidizing multiple signaling molecules (Mahadev et al., 2001, 2004; Loh et al., 2009; Cheng et al., 2010), while long-term excessive ROS may cause insulin resistance (Szendroedi et al., 2012).

Metabolic imbalance attributed to DIO has been shown to disrupt the structure and function of the olfactory system. When mice were challenged with high-fat or high-fat, high-carbohydrate diets, the excess energy imbalance resulted in marked loss of olfactory sensory neurons (OSNs), loss of axonal projections to defined glomerular targets, reduced electro-olfactogram amplitude, irregular AP firing frequency in mitral cells of the OB, reduced olfactory discrimination ability, slowed reward-reinforced behaviors, and disrupted reversal learning (Fadool et al., 2011; Thiebaud et al., 2014). Insulin-induced Kv1.3 phosphorylation in the OB (Fadool and Levitan, 1998; Fadool et al., 2000) was lost in DIO mice (Marks et al., 2009) and slices from these mice exhibited loss of insulin modulation of mitral cell excitability, exemplifying a degree of insulin resistance for mitral cell function in the obese state (Fadool et al., 2011). Obesity-resistant Kv1.3^−∕−^ mice exhibited similar loss of OSNs to that of WT mice while challenged with high-fat, high-carbohydrate diet, but not when fed a high-fat diet. The Kv1.3^−∕−^ mice showed a concomitant loss of olfactory discrimination and slowed reward-reinforced behaviors only when challenged with the high-fat, high-carbohydrate diet. Kv1.3^−∕−^ mice oddly showed improved olfactory discrimination with the high-fat diet and showed reversal learning capacity regardless of diet.

Herein, in an effort to further elucidate the mechanism of the metabolic phenotype of Kv1.3^−∕−^ mice, we report the presence of GLUT4 in the same OB neurolamina as the Kv1.3 channel and explore the ultrastructure of mitochondria in the Kv1.3^−∕−^ mice.

## Materials and methods

### Ethics statement

All animal experiments were approved by the Institutional Animal Care and Use Committee (IACUC) at the Florida State University (FSU) under protocols #1124 and #1427, and followed the guidelines set forth by the National Institutes of Health (NIH) and the American Veterinary Medical Association (AVMA). Mice were anesthetized with isoflurane (Aerrane; Baxter, Deerfield, IL, USA) using the IACUC-approved drop method and were then sacrificed by decapitation (AVMA Guidelines on Euthanasia, June 2007).

### Solutions

Phosphate-buffered saline (PBS) contained (in mM): 136.9 NaCl, 2.7 KCl, 10.1 Na_2_HPO_4_, and 1.8 KH_2_PO_4_ (pH 7.4). Phosphate-buffered saline + Tween (PBST) contained 1x PBS and 0.1% Tween. Hybridization buffer contained 0.5 M formamide, 0.25 M 20x SSC, 5 mg/mL torula yeast RNA, 50 mg/mL heparin sodium salt, and 0.1% Tween. 20x SSC contained 3 mM sodium chloride and 342 μM sodium citrate (pH 7.2). Blocking solution for *in situ* hybridization contained 0.2% BSA in PBST. NTMT contained (in mM): 100 Tris-HCl (pH 9.5), 25 MgCl_2_, 100 NaCl, and 0.1% Tween 20 (Cold Spring Harbor Protocols; http://cshprotocols.cshlp.org/). Wash buffer contained (in mM); 25 Tris, 250 NaCl, 5 EDTA, and 0.1% Triton X-100 (pH 7.5). Phosphatase inhibitor mixture (PPI) contained 1 mM phenylmethylsulfonyl fluoride, 10 μg/mL aprotinin, 1 μg/mL leupeptin, and 1 μg/mL pepstatin. All salts and reagents were obtained from Sigma Chemical (St Louis, MO, USA) or Fisher Scientific, Inc. (Atlanta, GA, USA) unless otherwise stated.

### Animal care

All mice used in our study were adult C57BL/6J male mice (000664 | Black 6; The Jackson Laboratory, Bar Harbor, ME, USA). Mice were weaned at postnatal day 29 and singly housed in conventional open cages at the FSU vivarium under a 12/12 h light/dark cycle with *ad libitum* access to food and water. For ultrastructural studies, wild-type (WT) and Kv1.3^−∕−^ mice (Xu et al., 2003; Fadool et al., 2004) were maintained on either a control diet (CF; Purina 5001 Rodent Chow; 28.05% kcal protein, 59.81% kcal carbohydrate, and 13.5% kcal fat; Richmond,VA, USA) or a moderately high-fat condensed milk diet (MHF; Research Diets D12266B; 16.8% kcal protein, 51.4% kcal carbohydrate, and 31.8% kcal fat; New Brunswick, NJ, USA) for a duration of 4 months.

### *In situ* hybridization

#### RNA probes

RNA from the OB of WT and Kv1.3^−∕−^ mice was isolated with Trizol reagent (Life Technologies, CA, USA). Total RNA was phenol/chloroform extracted, precipitated with isopropanol, washed with ethanol, and then dissolved in ultra-pure distilled water. Total cDNA was obtained by reverse transcription with oligo dT primer (Invitrogen, Carlsbad, CA, USA). Targeted genes were amplified by polymerase chain reaction using the following primers: GLUT4 primers forward 5′-TCTCAGCTGCCTTCCGAC-3′ and reverse 5′-TACCAAAGGCTCCCTCCC-3′; Kv1.3 primers forward 5′-CTTCGACGCCATCCTCTACTAC-3′ and reverse 5′-AAGCTCAAAGGAGAACCAGATG-3′; TH primers forward 5′-GATTGCAGAGATTGCCTTCC-3′ and reverse 5′-CCTGTGGGTGGTACCCTATG-3′. Kv1.3 and GLUT4 amplicons were cloned into pGEMT (Promega, Madison, WI, USA). The vector was linearized with *Sac* I for Kv1.3 and *Xba* I for GLUT4. Digoxigenin-UTP labeled RNA probes were made according to the manufacturer's specifications (Roche, Indianapolis, IN, USA) using SP6 RNA polymerase. Tyrosine hydroxylase (TH) RNA probe was generated as a positive control (result not shown) (Cubells et al., 1995), and Kv1.3 probe was applied to slices of Kv1.3^−∕−^ mice as a negative control.

#### In situ hybridization

WT and Kv1.3^−∕−^ mice were perfused with 4% paraformaldehyde (PFA), and the OBs were quickly dissected and placed in the perfusion solution overnight at 4°C. Thirty- micron slices were cut on a microtome (Leica CM1850, Buffalo Grove, IL, USA) and were left free floating in 100% methanol until use. For labeling, free-floating slices were transferred to a nine-well glass plate. Slices were first re-hydrated in a graded methanol series diluted in PBS (75, 50, and 25%) for 5 min each then 4 times with 100% PBST 0.1% for 5 min each. Slices were digested with 10 mg/mL proteinase K dissolved in PBST for 15 min, followed by a 10-min rinse in PBST, a 20-min fixation with PFA, and five rinses of 5 min each in PBST. Slices were pre-hybridized in hybridization buffer for 2 h at 60 °C, and then hybridized overnight with the probe at 60°C. Probe concentration was 0.5 ng/μL and total volume applied was 300 μL. The next day, slices were first rinsed at 60°C with graded dilutions of 2x SSC in hybridization buffer (25, 50, 75, and 100%) 10 min each, then twice at 68°C with 100% 0.2x SSC for 30 min, followed by 5-min rinses with graded dilutions (75, 50, 25, and 0%) of 0.2x SSC in PBST. PBST was then removed and replaced with blocking solution for 2 h at 4°C. Anti-digoxigenin Fab fragment conjugated to alkaline phosphatase was added overnight at 4°C. On day 3, slices were washed with blocking buffer then NTMT. DIG-labeled RNA probes were immunodetected with DIG Nucleic Acid Detection Kit (NBT/BCIP) according to the manufacturer's protocol (Roche). Slices were finally mounted on microscopic slides before visualization.

### Electron microscopy

All electron microscopy reagents were purchased from Electron Microscopy Sciences (EMS; Hatfield, PA, USA) unless otherwise noted. Following maintenance on modified diets, mice were perfused through the heart with PBS then with 4% PFA. OBs were quickly dissected and fixed in 4% PFA overnight, followed by infiltration with 10, and 30% sucrose in PBS at 4°C. Coronal OB sections were cut to 100 μm thickness on a vibratome (Leica Model 1000, Wetzlar, Germany), and transferred to a 24-well plate. Slices were washed three times with PBS, and then were incubated in 2% osmium tetroxide at room temperature for an hour. This was followed by a series of graded alcohol washes with 50% ethanol (EtOH) and 70% EtOH for 10 min each. The sections were further incubated with 1% uranyl acetate in 70% EtOH for 1 h before being dehydrated sequentially in 70, 90 and twice in 100% EtOH for 10 min each. After dehydration, sections were washed twice in propylene oxide for 10 min after which they were embedded in EMbed 812 Resin /propylene oxide (1:1) overnight on a rotary shaker in a fume hood to allow the propylene oxide to gradually evaporate. The following day, sections were infiltrated twice with fresh Epon for 12 h each on a shaker. Next the sections were flat embedded in fresh Epon between ACLAR plastic sheets (EMS) and polymerized in a 60°C oven over 3 days. ACLAR sheets were examined with a Nikon SMZ1000 stereoscopic microscope (Melville, NY, USA) and areas of the Epon film containing stretches of intact OB tissue layers were excised and remounted on Epon blocks with Scotch Super Glue Liquid (3M). Silver-gold sections (90–100 nm) were cut with a Leica Ultracut E microtome (Buffalo Grove, IL, USA) and mounted on 3.05 mm copper hexagonal mesh grids. The grids were post-stained with 2% uranyl acetate in ddH_2_O for 10 min followed by 4% Reynold's lead citrate for 5 min. Images were recorded on a FEI CM120 transmission electron microscope (Hillsboro, OR, USA) using a Tietz Tem-Cam F224 slow scan CCD camera. Mitochondrial counts were taken from a series of low magnification electron micrographs totaling ~2000 μm^2^ for each experimental condition. Within each 51.5 μm^2^ field of view, all mitochondria were counted. Mitochondrial area and circularity (4π × area / perimeter^2^) were quantified using Photoshop CS4 software (Adobe, San Jose, CA, USA). Approximately 40 fields of view from each experimental condition (CF^+∕+^, MHF^+∕+^, CF^−∕−^, MHF^−∕−^) were analyzed as sampled from 3 mice in each condition.

### cDNA constructs

The expression vectors were mammalian-based and contained the cytomegalovirus (CMV) promotor upstream from the coding region. Kv1.3 was ligated into the Invitrogen vector pcDNA_3_ at the unique *Hind* III site of the multiple coding region as previously described (Fadool et al., 2000). A non-conducting mutant Kv1.3 channel (W386F Kv1.3) was generated by site-directed mutagenesis as previously described (Holmes et al., 1997). The cDNA for GLUT4-myc-eGFP was generously provided by Dr. Jeffrey E. Pessin (Albert Einstein College of Medicine, Bronx, NY, USA).

### Cell maintenance and transient transfection

Human embryonic kidney (HEK) 293 cells were handled according to the guidelines of the European Center for the Validation of Alternative Methods Task force based on the Guidance on Good Cell Culture Practices (GCCP; Coecke et al., 2005). Cells were grown at 37°C in modified Eagle's minimum essential media (MEM; Gibco/Life Technologies, Carlsbad, CA, USA) supplemented with 2% penicillin/streptomycin (Sigma-Aldrich) and 10% fetal bovine serum (Gibco/Life Technologies). Cells were grown to 75–80% confluency for 3–4 days, after which cDNA was introduced with lipofectamine (Thermo Fisher Scientific, #18324-012; Waltham, MA, USA) as previously described (Marks and Fadool, 2007). Briefly, 3 μg of each cDNA construct was complexed with 18 μl of lipofectamine for 30 min in Opti-MEM (Gibco), after which the cDNA/lipofectamine/Opti-MEM mixture was applied to HEK293 cells in a 60 mm dish for 5 h before the transfection was stopped by replacing with MEM. Proteins were immunoprecipitated 2 days post transfection.

### Immunoprecipitation and western blot

Cells were lysed and prepared for immunoprecipitation following a modification of procedures by Colley et al. (2007). Two days post transfection, HEK 293 cells designated for detection of surface GLUT4 were first incubated for 30 min at 37°C with 1:300 c-myc antibody (9E10 clone, Roche), then washed three times with sodium orthovanadate diluted in PBS. Cells were then lysed in ice-cold PPI mixture for 30 min. The lysates were clarified by centrifugation at 14,000 g for 10 min at 4°C and the supernatant rotated overnight at 4°C on a Roto-Torque slow speed rotary (model 7637; Cole Palmer, Vernon Hills, IL). The immunoprecipitated protein harvested the next day with 3 mg/ml Protein A-Sepharose (GE Healthcare, Pittsburgh, PA, USA). To collect total GLUT4, cells were lysed first with PPI and then precleared with protein Sepharase A for 3 h before anti-myc (1:400; 9E10 clone, Roche) was added overnight to the whole-cell lysate. The next day, protein Sepharose A was added again to the immunoprecipitated proteins for 3 h, followed by 4 wash steps using centrifugation at 14,000 g (4°C) for 10 min each with wash buffer (see section Solutions).

Immunoprecipitated proteins from whole-cell or surface detection of GLUT4 were separated on 10% acrylamide sodium dodecyl sulfate (SDS) gels and electro-transferred to nitrocellulose for Western blot analysis as previously described (Colley et al., 2007). Blots were blocked with 1% nonfat dry milk (Biorad Laboratories, Hercules, PA, USA), and incubated overnight at 4°C in primary antibody against c-myc (1:700) then with secondary antibody (goat anti-mouse; 1:3000; Sigma A2304) for 90 min at room temperature. Protein bands were visualized using enhanced chemiluminescence Western blotting detection reagent (ECL; GE Healthcare) exposure on Fuji RX film (Fisher Scientific). A Hewlett-Packard Photosmart Scanner (model 106–816, Hewlett Packard, San Diego, CA) was used in conjunction with Quantiscan software (Biosoft, Cambridge, England) to quantify the densitometry of protein bands revealed on the film autoradiographs.

### Statistical analysis

All data are reported as the mean (± s.d.). Significantly-different means were determined by a one-way analysis of variance (ANOVA) due to multiple comparison design of all experiments. A Bonferoni *post-hoc* test was applied for protein biochemistry experiments and a Student Newman Kuels (*snk*) was applied for electron microscopy experiments. Different length vertical lines represent significantly different *post-hoc* comparison.

## Results

### mRNA of GLUT4 and Kv1.3 are co-localized in mitral cells of the olfactory bulb

**Figure 1:**
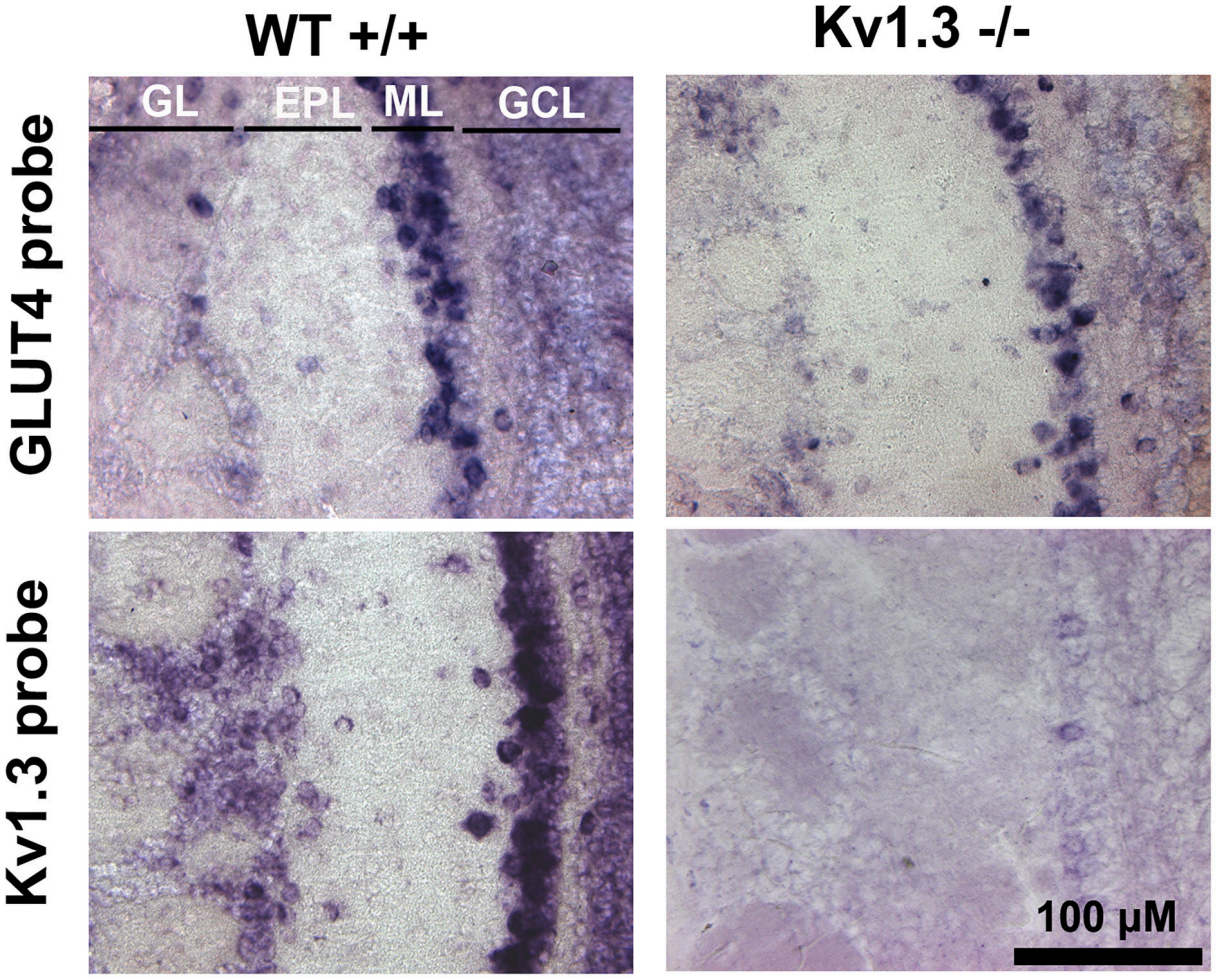
**Kv1.3 and GLUT4 mRNA are expressed predominantly in the glomerular and mitral cell layers of the olfactory bulb**. Light photomicrographs containing tissue sections of wildtype (WT^+∕+^; left) and Kv1.3-null mice (Kv1.3^−∕−^; right) olfactory bulbs labeled with GLUT4 (GLUT4 probe; top) or Kv1.3 (Kv1.3 probe; bottom). Note that GLUT4 mRNA is expressed predominantly in the mitral cell layer (ML) in both WT and Kv1.3^−∕−^ mice, whereas Kv1.3 mRNA is localized to both the glomerular layer (GL) and ML. GL, glomerular layer; EPL, external plexiform layer; ML, mitral cell layer; GCL, granule cell layer.

We have previously demonstrated that mitral cells are glucose-sensitive and that modulation of AP firing frequency is dependent upon Kv1.3 (Tucker et al., 2010, 2013). GLUT4 mRNA is expressed in 75% of glucose-sensing neurons of the ventromedial nucleus of the hypothalamus (Kang et al., 2004). We therefore hypothesized that GLUT4 would be localized to the mitral cell layer where Kv1.3 channels carry 60–80% of the outward current in these primary output neurons (Fadool and Levitan, 1998). mRNA expression of GLUT4 was examined by *in situ* hybridization with digoxigenin-labeled riboprobes. A robust expression of GLUT4 transcripts was found in the soma of mitral cells within the mitral cell layer (ML) from WT mice (Figure 1). A faint labeling was observed in the granule cell layer (GCL) and a few periglomerular cells (PG) expressed GLUT4 mRNA. The expression pattern for GLUT4 was similar in Kv1.3^−∕−^ mice, suggesting that the loss of Kv1.3 channel did not affect transporter transcript expression. The Kv1.3 mRNA transcript was also robustly visualized in the ML of WT mice in nearly every mitral cell that was examined. Kv1.3 mRNA was also visualized in PG cells to a greater extent than that of the GLUT4 probe. An absence of Kv1.3 mRNA labeling was observed in Kv1.3^−∕−^ across all neural lamina (Figure 1, bottom). A similar absence of labeling was found for slices where the probes were omitted (data not shown).

### Glut4 translocation is facilitated by blocking kv1.3 conductance

**Figure 2:**
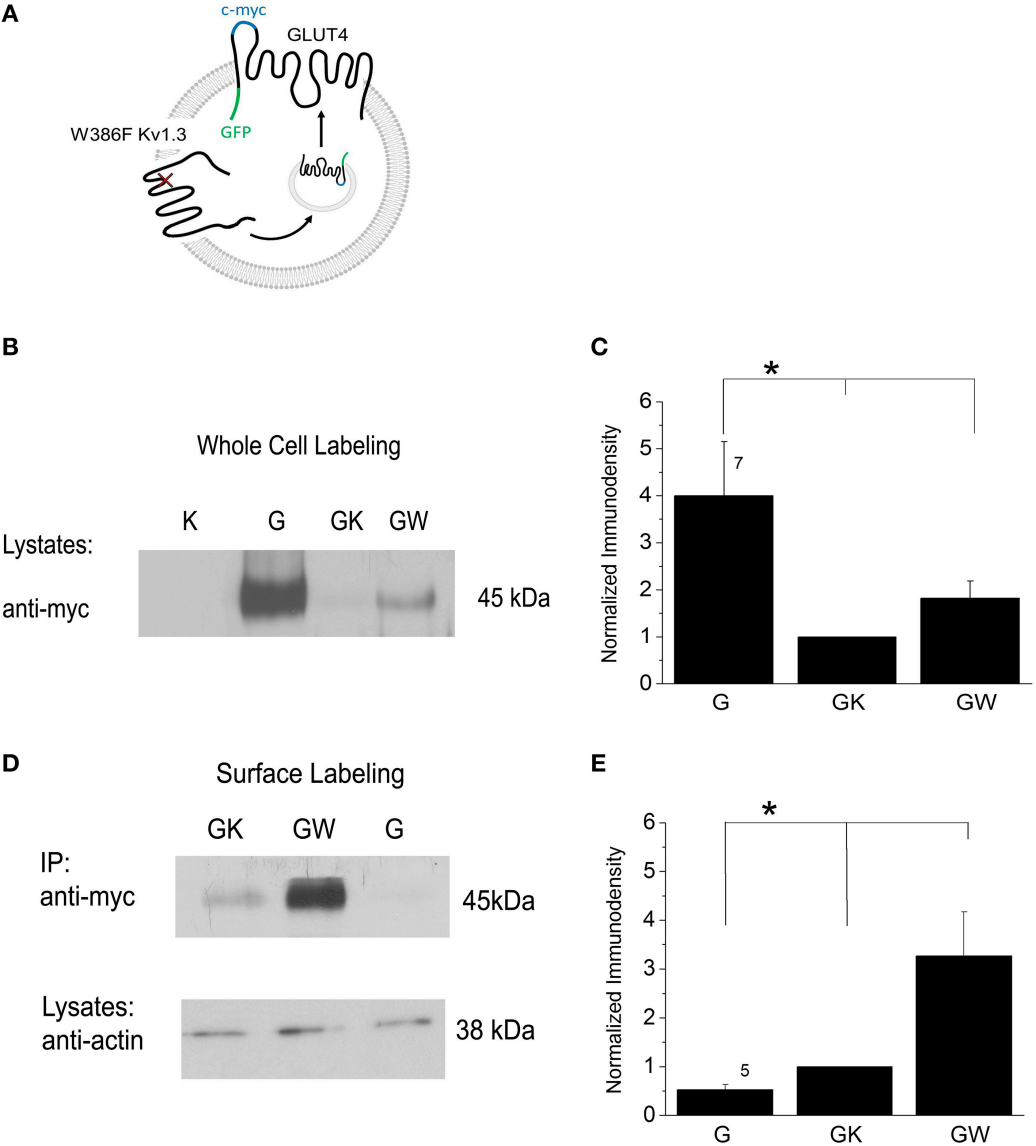
**GLUT4 is expressed intracellularly and then translocates to the plasma membrane following block of Kv1.3 channel achieved by site-directed mutagenesis of the pore. (A)** Model of heterologous co-expression of a non-conducting Kv1.3 channel (W386F Kv1.3) and an extracellular epitope-myc tagged GLUT4 transporter that becomes translocated to the plasma membrane. **(B)** Western blot of lysates harvested from HEK293 cells transfected with K: Kv1.3 cDNA; W: mutant W386F Kv1.3 cDNA; G: GLUT4-myc cDNA. Blots were probed with myc antibody (anti-myc) to visualize labeling of all GLUT4 in a whole-cell preparation. **(C)** Bar graph of the normalized pixel immunodensity of the quantified bands for 7 such blots as represented in **(B)**. ^*^ = one-way analysis of variance (ANOVA), Bonferoni *post-hoc* test, *p* ≤ 0.05. Different length vertical lines represent significantly different *post-hoc* comparison. **(D,E)** Same as **(B,C)** but samples were surface immunoprecipitated (IP) with myc antibody (anti-myc) and then labeled with anti-myc to visualize labeling of surface GLUT4. Input (lysates) were labeled with actin antibody (anti-actin) to standardize equal loading.

Mice with genetic deletion of Kv1.3 are resistant to modulation by glucose (Tucker et al., 2013). Kv1.3 serves as an insulin receptor (IR) kinase substrate, and its activity can be inhibited by tyrosine phosphorylation (Fadool et al., 2000). Xu et al. (2004) found that genetic or pharmacological blockade of Kv1.3 exerted the same effect as insulin whereby GLUT4 translocation to the membrane of adipocytes and skeletal muscles was increased. Kv1.3 inhibition enhances GLUT4 translocation in adipocytes through a PI3K-independent mechanism consisting of depolarizing the plasma membrane and increasing intracellular Ca^2+^ concentration (Li et al., 2006). To prove that Kv1.3 activation serves as molecular trigger of GLUT4 translocation to the membrane, HEK 293 cells were transiently transfected with the cDNA coding for GLUT4 plus wildtype or a pore mutant Kv1.3 channel. W386F Kv1.3 is a construct whereby a threonine is substituted for a phenylalanine and results in the translation of a non-conducting channel (Holmes et al., 1997; Delaney et al., 2014). Whole-cell or surface-labeled GLUT4 was visualized using anti-c-myc directed against a myc extracellular, epitope tag on GLUT4 (Figure 2A). Whole-cell lysates showed a strong label for total GLUT4 cell expression (Figures 2B,C). GLUT4 + Kv1.3 yielded a significantly lower total receptor cell expression that was increased when the W386F Kv1.3 was substituted in the transfection scheme [significantly different, one-way analysis of variance (ANOVA) with Bonferoni's *post-hoc* test, *F*_(2, 18)_ = 5.032, *p* = 0.0184]. Immunoprecipitation (IP) was used to isolate the surface-labeled GLUT4 from total receptor cell expression (Figures 2D,E). The IP strategy revealed very little GLUT4 at the plasma membrane during a static state, consistent with its known intracellular storage pool reported by Li et al. (2009). However, a consistent expression of GLUT4 was found in the Kv1.3 co-transfection condition and a strong increase in receptor expression was observed when Kv1.3 was substituted by W386F Kv1.3 [significantly different, one-way ANOVA with Bonferoni's *post-hoc* test, *F*_(2, 12)_ = 6.242, *p* = 0.0139]. Our results indicate that changes in Kv1.3 conductance produced by pore mutagenesis upregulates GLUT4 translocation to the membrane surface. Such regulated receptor membrane availability would reflect the capacity to modify glucose utilization at any given time.

### Kv1.3^−∕−^ mice have smaller mitochondria that are resistant to morphological changed induced by diet-induced obesity

Elevated plasma glucose after a meal activates mitochondrial oxidative phosphorylation in pancreatic cells to increase ATP release and insulin secretion. A chronic rise in glucose, such as in diabetic or obese patients, has caused mitochondrial atrophy, which suggests an important connection between mitochondria and metabolic imbalance (Gerbitz et al., 1996). Several potassium channels contribute to mitochondrial function including Kv1.3 (Bednarczyk, 2009). We decided to investigate the change in mitochondrial morphology in the OB of WT and Kv1.3^−∕−^ mice that have been challenged with a MHF vs. CF diet.

**Figure 3:**
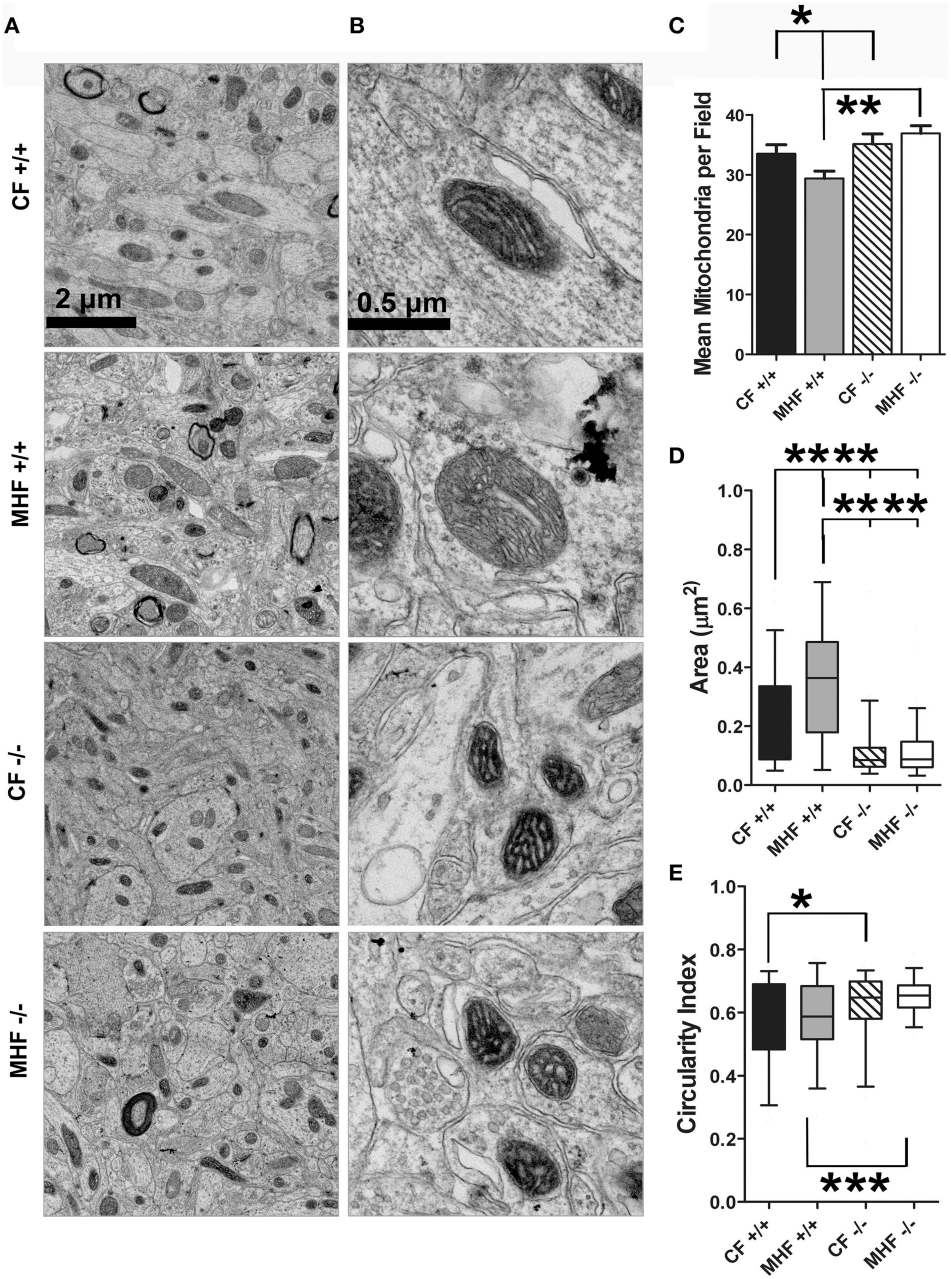
**Kv1.3^−∕−^ mice have smaller mitochondria in the mitral cell layer of the OB, which do not exhibit an increase in volume when challenged with moderately-high fat diet (MHF)**. Photomicrographs acquired from the mitral cell layer of the OB of wildtype (+/+) and Kv1.3-null mice (–/–) maintained on MHF vs. CF for 4 months. **(A)** Low and **(B)** higher magnification. Bar graph or box blot representing **(C)** mean number, **(D)** cross-sectional area, and **(E)** circularity index of mitochondria collected across 40 fields of view for 3 mice. Data represent mean +∕− s.d. for the bar graph, or mean (line), 25/75% quartile (box) and 5/95% (whiskers) for the box plot. Significantly different, one-way ANOVA, Bonferoni's *post-hoc* test, ^****^p ≤ 0.0001; ^***^p ≤ 0.001, ^**^p ≤ 0.01; ^*^p ≤ 0.05.

Electron micrographs showing the abundance and size of mitochondria examined within the cell bodies of mitral cells are found in Figures 3A,B, respectively (low and higher magnification). The number, cross-sectional area, and circularity index of mitochondria from 40 fields of view were quantified from three mice in each of the four conditions (Figures 3C–E). For control fed mice, there was no change in the number of mitochondria across the genotypes [number: one-way ANOVA, *F*_(3, 161)_ = 4.925, *p* = 0.0027, Student Newman Keuls's (*snk*) *post-hoc* test CF^+∕+^ vs. CF^−∕−^, *p* ≥ 0.05], however the cross-sectional area was larger and circularity index was less in the WT mice compared with that of the Kv1.3^−∕−^ mice [area: one-way ANOVA, *F*_(3, 305)_ = 51.36, *p* ≤ 0.0001, *snk post-hoc* test CF^+∕+^ vs. CF^−∕−^ significantly different, *p* ≤ 0.0001; circularity index: one-way ANOVA, *F*_(3, 302)_ = 8.178, *p* ≤ 0.0001, *snk post-hoc* test CF^+∕+^ vs. CF^−∕−^ significantly different, *p* ≤ 0.05]. When WT mice were challenged with a MHF diet, the mitochondrial cross-sectional area but not the circularity index became enlarged [area: one-way ANOVA, *F*_(3, 305)_ = 51.36, *p* ≤ 0.0001, *snk post-hoc* test CF^+∕+^ vs. MHF^+∕+^ significantly different, *p* ≤ 0.0001; circularity index: one-way ANOVA, *F*_(3, 302)_ = 8.178, *snk post-hoc* test CF^+∕+^ vs. MHF^+∕+^, *p* ≥ 0.05] and the abundance of mitochondria decreased [number: *F*_(3, 161)_ = 4.925, *p* = 0.0027, *snk post-hoc* test CF^+∕+^ vs. MHF^+∕+^ significantly different, *p* ≤ 0.05]. In contrast, a MHF-diet challenge did not affect mitochondria number, area, or circularity in the Kv1.3^−∕−^ mice (one-way ANOVA, *snk post-hoc* test, *p* ≥ 0.05). These data provide evidence that diet high in fat changes the morphology of mitochondria in the OB, and that gene-targeted deletion of Kv1.3 precludes these effects.

## Discussion

The voltage-gated *Shaker* potassium channel Kv1.3 is a key contributor in regulating body weight, metabolism, and energy homeostasis. Fewer data are reported elucidating specific potential molecular mechanisms linking Kv1.3 kinetics and energy balance. Herein we provide new evidence for co-localization of Kv1.3 and the glucose transporter type 4 (GLUT4) in the mitral cell layer of the OB. We further demonstrate that GLUT4 is a possible substrate for Kv1.3 activation whereby functional Kv1.3 increases GLUT4 expression on the membrane and Kv1.3 current suppression strongly upregulates GLUT4 transport from intracellular storage to the plasma membrane. Additionally, we show that gene-targeted deletion of Kv1.3 channel changes mitochondrial shape and reduces their size. Moreover, WT mice that are challenged with a MHF-diet exhibit a decrease in number of mitochondria within the mitral cells of the OB whereas those from Kv1.3^−∕−^ mice do not demonstrate such loss. Mitochondria within Kv1.3^−∕−^ mice are also resistant to increase in size following MHF-challenge, which is observed in WT mice.

**Figure 4:**
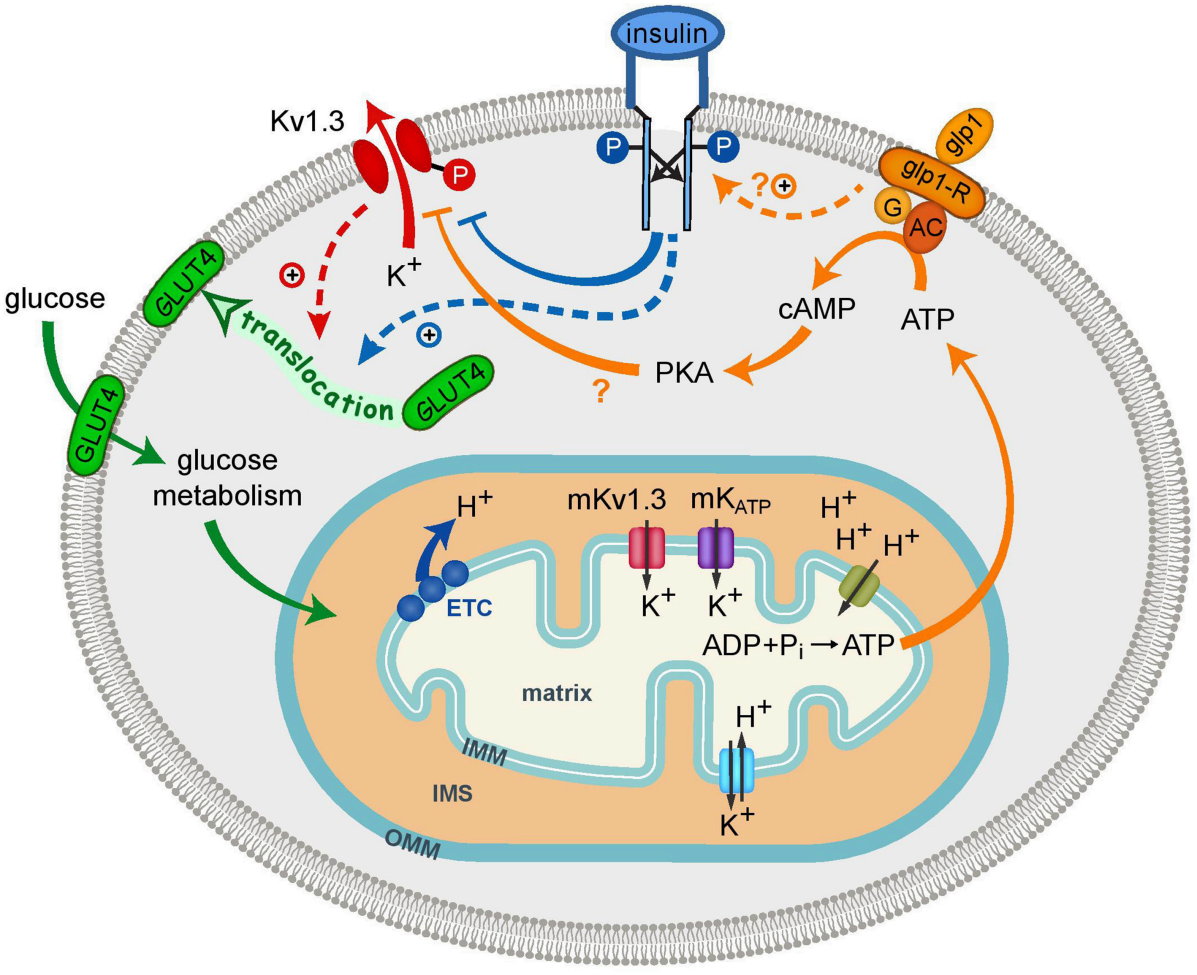
**Schematic diagram showing cell signaling interactions of plasma membrane Kv1.3 (Kv1.3) and potential interplay with mitochondrial Kv1.3 (mKv1.3)**. Glycogen-like peptide (GLP-1) and insulin are two signaling hormones that block Kv1.3 current. The channel (red) is known to be a substrate for insulin receptor (IR) kinase (blue) on the N- and C-terminal aspects of the channel protein (Y111-113, Y137, Y479). Insulin-induced phosphorylation (P) of Kv1.3 (blue line) decreases Kv1.3 current amplitude by decreasing Pr_open_ of the channel. GLUT4 is typically translocated to the membrane upon insulin activation (blue dashed line) but upon block of Kv1.3 conductance, can also be translocated (red dashed line). Metabolism of glucose establishes the H^+^ gradient and the production of ATP via the electron transport chain (ETC). Maintenance of the ionic environment is dependent upon influx of K^+^ through mKv1.3 and mKATP and the balance provided by efflux via the K^+^/H^+^ exchanger. The ATP energy source could be used for the GLP-1R triggered conversion of ATP to cAMP through adenylase cyclase to increase potential PKA activity (?). Metabolic factors that decrease Kv1.3 ion channel activity increase the AP firing frequency in mitral cells that is thought to provide odor quality coding of olfactory information. OMM, outer mitochondria membrane; IMM, inner mitochondria membrane; IMS, inter membrane space.

As summarized in Table 1, GLUT4 is expressed in several discrete brain regions such as the hypothalamus, the hippocampus, the cerebellum, and the amygdala. GLUT4 in the brain is mainly neuronal, and localizes on dendrites or on the membranes of transport vesicles, Golgi apparatus, and the rough endoplasmic reticulum (Kobayashi et al., 1996; Leloup et al., 1996; McCall et al., 1997; El et al., 1998; Choeiri et al., 2002). In the OB, GLUT4 proteins are localized on the dendritic terminals of mitral/tufted cells at the level of glomeruli, which are sites of synaptic connectivity between the axons of OSNs and mitral cells, and in the nuclei of periglomerular cells (Sharp et al., 1975). Our *in situ* hybridization results indicate that GLUT4 and Kv1.3 transcription occurs in the cell bodies of mitral cells and interneurons before being translated and recruited at the synapse for glucose-based modulation of olfactory information. Interestingly, Sharp and collaborators also found that GLUT4 protein was co-localized with IR on mitral cells (Sharp et al., 1975, 1977). This is highly consistent with our characterization of IR modulation of Kv1.3 via tyrosine phosphorylation on the N- and C-terminal aspects of the channel, and our finding of co-localization of the channel with the kinase in mitral cells (Fadool et al., 2000). Because our previous electrophysiological data demonstrate nearly all mitral cells are insulin sensitive (Fadool et al., 2011), combined with our current gene expression data and the protein data from the Sharp laboratory (Sharp et al., 1975, 1977), it is highly plausible that all three signaling molecules are contained within mitral cells (Figure 4). Although it remains to be shown if all mitral cells are homogeneous in their expression of Kv1.3/GLUT4/IR or whether there is a degree of heterogeneity across this population of first order neurons (Angelo et al., 2012; Padmanabhan and Urban, 2014), the modeled signaling interactions could influence glucose sensing, and concurrently, electrical signaling, by mitral cells. Decreased Kv1.3 activity evokes translocation of GLUT4 to the membrane to permit glucose uptake and the end production of ATP. In our model, we demonstrate two known modulators of Kv1.3 activity that are related to incretin hormones (glycogen-like peptide signaling and IR kinase), which decrease Kv1.3 conductance through either a confirmed (Fadool and Levitan, 1998; Fadool et al., 2000, 2011) or suspected (Thiebaud et al., 2016) phosphorylation (P) of the channel. Our mutagenesis data directly demonstrate that blocked conduction of Kv1.3 current is solely capable of GLUT4 translocation and this is consistent with earlier pharmacological experiments from the Desir laboratory that applied known potassium channel blockers to visualize surface expression of GLUT4 by immunocytochemistry (Xu et al., 2004). Because our HEK cell model expresses only a few components of the IR signaling cascade (Thomas and Smart, 2005) and our experiments were performed without the hormone insulin, our biochemical data demonstrate the ability of Kv1.3 and GLUT4 to interact without the necessity of insulin to either translocate GLUT4 or alternatively to phosphorylate Kv1.3. We conjecture that in native mitral cells, insulin could induce GLUT4 translocation either through direct IR activation or through Kv1.3 inhibition caused by IR phosphorylation (blue and red dashed lines, respectively; Figure 4). Our model could include downstream signaling well characterized in the periphery, however, the existence of these parallels in the CNS is not known. For example, Kv1.3 inhibition enhances GLUT4 translocation in adipocytes through a PI3K-independent mechanism consisting of depolarizing the plasma membrane and increasing intracellular Ca^2+^ concentration (Li et al., 2006). GLUT4 translocation to the membrane is classically facilitated by insulin binding to its receptor, which triggers PI3K-dependent or -independent pathways (Hoffman and Elmendorf, 2011). Another interesting caveat is that GLUT4 expression in the OB seems to be modulated by the feeding state. Postprandially when glucose levels in the blood are high, GLUT4 is expressed on the plasma membrane of dendritic processes. GLUT4 in fasted rats however, is internalized into the cytoplasm (Al Koborssy et al., 2014) even though insulin levels in the OB are shown to be 15 fold higher in the fasting state (Fadool et al., 2000). Our biochemical data point to a role of Kv1.3 in controlling GLUT4 expression on the membrane but more evidence is needed to prove that this modulation could bypass IR kinase *in vivo*.

Because Kv1.3^−∕−^ mice have an increased locomotor activity and metabolism, a change in mitochondrial shape might be anticipated. Our ultrastructural data demonstrate that control fed Kv1.3^−∕−^ mice have an altered circularity shape compared with similarly fed WT mice. Additionally, the cross-sectional area of the mitochondria is smaller in the Kv1.3^−∕−^ regardless of dietary treatment. Neuronal mitochondria typically have cristae that form a network of anastomosing tubes and the number of cristae is correlated with the level of aerobic activity and associated ATP production (Lehninger, 1982). While our analysis did not quantify this sub-organelle structure, qualitatively the cristae from the WT mice appeared to be more longitudinally arranged. During high rates of metabolism, the mitochondrial membranes can become condensed due to structural changes of the IMM and matrix, as opposed to orthodox or “idling” mitochondria that are not actively respiring (Lehninger, 1982). This type of shrinking does not appear to be the cause of smaller mitochondria in the Kv1.3^−∕−^ because the outer mitochondrial membrane (OMM) is observed closely juxtaposed to the IMM and cristae. Future functional studying of mitochondria from Kv1.3^−∕−^ mice are needed to test the idea that the more condensed mitochondrial volume might contribute to increased TEE. Interestingly in the rat OB, the respiratory control ratio that measures mitochondrial activity is 2x higher that in the hypothalamus (Benani et al., 2009) and is in accordance with the high energy demand of the glomeruli (Nawroth et al., 2007). What is also of interest for mitochondria in the OB is the high expression of GLUT4 in this region and the total energy budget for olfactory discrimination. Odor-evoked oxidative metabolism of OB synaptic transmission is energetically demanding and is tightly correlated to capillary density (Lecoq et al., 2009).

The challenge of a MHF-diet did not decrease the number or increase cross-sectional area of mitochondria in the Kv1.3^−∕−^ mice as it did in the WT mice. This is very intriguing given that mitochondria are highly dynamic in morphology and abundance. In liver, short-term challenge (21 day) with a high-fat diet causes a decrease in proteins of the electron transport chain and transporters/exchangers of the IMM, but does not cause a change in mitochondrial volume (Kahle et al., 2015). The influx of potassium across the IMM (Figure 4) through both mKv1.3 and mK_ATP_ could cause an increase in volume changes of the mitochondria if not tightly counterbalanced by the H^+^/K^+^ antiport exchanger (Garlid, 1996). One role of the H^+^/K^+^ antiport exchanger is to counterbalance the influx of K^+^ that allows the high membrane gradient required for oxidative phosphorylation and thereby acts to provide volume homeostasis (Garlid and Paucek, 2003). In the absence of Kv1.3, there may be less K^+^ influx, leading to smaller mitochondria. In the condition of long-term (4 months) MHF-diet and precipitated DIO, the WT mice could have dysfunction in IMM ion transport resulting in increased volume or swelling. Such ionic imbalance could lead to unhealthy mitochondria, which might undergo mitophagy and lead to a loss in mitochondrial density or abundance such as we observed. The subsequent lack of volume change or loss of number in the fat-fed Kv1.3^−∕−^ mice could be either attributed to less initial K^+^ influx or changed fatty-acid metabolism that is also linked to organelle volume (Garlid, 1996) and would be anticipated to be reduced in the thin, obesity resistant mice.

Whether mitochondrial dysfunction is linked to glucose intolerance and insulin resistance is a topic of intensive research and current debate (see review, Montgomery and Turner, 2015) but certainly a greater number of mitochondria permit greater ATP production and a reduced number of mitochondria are found during periods of quiescence. Typical to what we observed in the DIO wildtype mice, a reduced number of mitochondria was associated with larger individual organelles. Although our ultrastructural data do not allow us to discern if the larger mitochondria had impaired function, by deduction, the fatty diet either decreased mitochondrial biogenesis or increased mitophagy to yield a decreased mitochondrial density in the obese mice. The fact that loss of Kv1.3 channel alters mitochondria shape and prevents increased volume of mitochondria when challenged with MHF suggests an alteration of normal dynamics of the organelle and associated fission and fusion proteins. Similar effects of high-fat diet on mitochondrial function have been observed in the central nervous system (Petrov et al., 2015). For example, Parton et al. (2007) attributed loss of glucose sensing by pro-opiomelanocortin (POMC) neurons of the hypothalamus in DIO mice to the mitochondrial uncoupling protein 2 (UCP2). Genetic deletion or pharmacological inhibition of UCP2 was able to restore glucose sensing in the obese mice, underscoring the role of mitochondrial dysfunction and altered ATP production in DIO.

One of the earliest changes in the development of insulin resistance attributed to DIO is ectopic lipid accumulation (Turner et al., 2013). In response to excess lipid availability in the wildtype DIO mice, increase reactive oxygen species (ROS) such as superoxides, hydroxyl radicals, and hydrogen peroxide could have been elevated (i.e., Paglialunga et al., 2015). Interestingly, Kv1.3 can be regulated by these types of second messengers. Phosphorylation of Kv1.3 on four serine residues by PKA and PKC is known to increase Kv1.3 activity to maintain a negative membrane potential (Chung and Schlichter, 1997a,b). As ATP is depleted below basal intracellular levels, the channel exhibits reduced peak current amplitude and a shift in voltage dependence. Application of ROS scavengers alleviates Kv1.3 phosphorylation to decrease the membrane potential (Cayabyab et al., 2000).

Plasma membrane bound Kv1.3 and mitochondrial Kv1.3 could influence or detect metabolic state at the level of the OB through channel neuromodulation and down-stream signaling cascades that could modify excitability. Insulin and the incretin hormone, GLP-1, can phosphorylate Kv1.3 at the plasma membrane, decrease Kv1.3 current and upregulate GLUT4 translocation to the membrane to facilitate glucose metabolism. In turn, the activity of mitochondrial Kv1.3 is linked to ROS and ATP production that regulate channel phosphorylation and conductance. Future targeted regulation of Kv1.3 in the OB could reveal the ability of this brain region to detect and regulate metabolic state by changing membrane excitability based on nutritional needs. The extent by which the mitral cell plasma and mitochondrial membrane activity interplays in detecting the chemistry of metabolism and olfactory coding utilizing Kv1.3 is a novel future trajectory in understanding brain energy sensing and dysfunction following obesity.

## Author contributions

DF conceived of the experiments; JF and DK conducted the *in situ* hybridization experiments; KK and JF conducted the electron microscopy (EM) experiments; DF, BC, and KK conducted the biochemistry experiments; DK and ZH analyzed the biochemistry and EM results respectively; all authors drafted the manuscript and critically evaluated the final content.

## Funding

This work was supported by NIH grant R01 DC013080 from the National Institutes of Deafness and Communication Disorders (NIDCD).

### Conflict of interest statement

The authors declare that the research was conducted in the absence of any commercial or financial relationships that could be construed as a potential conflict of interest.

## Abbreviations

AP: action potential
ATP: adenosine triphosphate
AVMA: American Veterinary Medical Association
BSA: bovine serum albumin
CF: control diet
CMV: cytomegalovirus
ddH_2_O: double-distilled water
DIO: diet-induced obesity
EtOH: ethanol
FSU: Florida State University
GCCP: Guidance on Good Cell Culture Practices
GCL: granule cell layer
GLUT4: glucose transporter type 4
h: hour
HEK: human embryonic kidney
IACUC: Institutional Animal Care and Use Committee
IMM: inner mitochondrial membrane
IP: immunoprecipitation
IR: insulin receptor
Kv1.3: voltage-dependent potassium channel 1.3
MEM: minimal essential medium
MHF: moderately high-fat diet
min: minute
ML: mitral cell layer
NADPH: nicotinamide adenine dinucleotide phosphate
NIH: National Institutes of Health
NTMT: alkaline phosphatase buffer
OB: olfactory bulb
OMM: outer mitochondrial membrane
OR: olfactory receptor
OSN: olfactory sensory neuron
PBS: phosphate-buffered saline
PBST: phosphate-buffered saline + Tween
PFA: paraformaldehyde
PG: periglomerular
PKA: protein kinase A
PKC: protein kinase C
PPI: phosphatase inhibitor
ROS: reactive oxygen species
SDS: sodium dodecyl sulfate
*snk*: Student Newman Keuls *post-hoc* test
SSC: saline sodium citrate
TEE: total energy expenditure
WT: wildtype.
